# Selective Laser Melting of Al-Based Matrix Composites with Al_2_O_3_ Reinforcement: Features and Advantages

**DOI:** 10.3390/ma14102648

**Published:** 2021-05-18

**Authors:** Ivan A. Pelevin, Anton Yu. Nalivaiko, Dmitriy Yu. Ozherelkov, Alexander S. Shinkaryov, Stanislav V. Chernyshikhin, Alexey N. Arnautov, Sergey V. Zmanovsky, Alexander A. Gromov

**Affiliations:** 1 Catalysis Lab, National University of Science and Technology MISIS, 119991 Moscow, Russia; i.pelevin@misis.ru (I.A.P.); nalivaiko@misis.ru (A.Y.N.); d.ozherelkov@gmail.com (D.Y.O.); shinkaryov@gmail.com (A.S.S.); aleksej.arnautov@gmail.com (A.N.A.); 2 Center for Design, Manufacturing and Materials, Skolkovo Institute of Science and Technology, 121205 Moscow, Russia; stanislav.chernyshikhin@skoltech.ru; 3 New Project Directorate, United Company RUSAL, 121096 Moscow, Russia; sergey.zmanovskiy@rusal.com

**Keywords:** metal matrix composites, selective laser melting, laser powder bed fusion, aluminum alloys, alumina, mechanical properties

## Abstract

Aluminum matrix composites (AMC) are of great interest and importance as high-performance materials with enhanced mechanical properties. Al_2_O_3_ is a commonly used reinforcement in AMCs fabricated by means of various technological methods, including casting and sintering. Selective laser melting (SLM) is a suitable modern method of the fabrication of net-shape fully dense parts from AMC with alumina. The main results, achievements, and difficulties of SLM applied to AMCs with alumina are discussed in this review and compared with conventional methods. It was shown that the initial powder preparation, namely the particle size distribution, sphericity, and thorough mixing, affected the final microstructure and properties of SLMed materials drastically. The distribution of reinforcing particles tends to consolidate the near-melting pool-edges process because of pushing by the liquid–solid interface during the solidification process that is a common problem of various fabrication methods. The achievement of an homogeneous distribution was shown to be possible through both the thorough mixing of the initial powders and the precise optimization of SLM parameters. The strength of the AMCs fabricated by the SLM method was relatively low compared with materials produced by conventional methods, while for superior relative densities of more than 99%, hardness and tribological properties were obtained, making SLM a promising method for the Al-based matrix composites with Al_2_O_3_.

## 1. Introduction

Metal matrix composites (MMCs) have become widely used as construction materials, among which Al-based MMCs are employed for highly demanding applications such as in aircraft and automotive industries, as well as the military [1,2,3,4,5,6,7]. The introduction of reinforcing particles into the metallic matrix is the admitted approach of economical materials production [8,9,10].

Superior mechanical properties, particularly high strength-to-weight ratios, ductilities, moduli, excellent wear and corrosion resistances, and creep resistances at elevated temperatures, could be obtained [5,11,12]. Parts of engines and pistons, etc. require superior tribological properties of the materials from which they are made, making aluminum matrix composites (AMCs) with Al_2_O_3_ or SiC reinforcing particles suitable for such applications [13,14,15,16,17,18,19,20,21,22]. The presence of the alumina particulates partially taking on external loads and exhibiting good bonding properties with the Al matrix provides an elevated wear resistance of the composite [23,24,25]. The feature to be considered as a rule is the reduced elongation of the composite [26,27,28]. Among reinforcements, Al_2_O_3_ benefits from its stability at elevated temperatures and dissolving into Al with no undesirable phase formations [29]. 

The aluminum alloy composites can be produced via various synthesis approaches [2,30], involving casting [31,32] and stir casting [5,33], which are characterized by a strong particle bonding, matrix structure control simplicity, and near-net-shape, as well as being one of the most economical [2,30,34,35,36]. These technological routes imply the addition of reinforcing particles into a molten matrix, and the main problems associated with casting processes are the achievements of sufficient wettability and a reinforcement phase heterogeneous distribution [33]. The difference between particles and matrix density leads to particles floating or sinking, thus causing a nonuniform dispersion [37,38,39,40]. If the density of alumina (3.97 g/cm^3^) is higher than that of aluminum (2.7 g/cm^3^), the sinking of particles is observed. The mixing of the matrix and ceramic particles in the solid state within powder metallurgy methods with subsequent sintering is considered the most widely used technique for aluminum alloy composites [4,41,42]. It allows the achievement of a better microstructure control with a more homogeneous reinforcement distribution. A significant difference in the melting points and compressive strength of alumina and aluminum interrupts the good rearrangement of the particles and leads to porosity, further complicating the Al-Al_2_O_3_ fabrication. It was observed that increasing alumina particle size leads to a higher porosity, whereas its decrease provides a higher hardness and tensile strength of the composite [4,5], which is attributed to a greater Al-Al_2_O_3_ interfacial area and to a higher likelihood of the fracture of large alumina particles [43]. The complex, expensive, and time- and energy-consuming routes such as mechanical alloying and diffusion bonding [36,44,45] are not considered in this review, because of their rare use. The common problems of all the methods include porosity and the emergence of particle clusters [33,46], which could drastically decrease the mechanical properties.

The modern approach for the fabrication of alloys and MMC includes Al-based composites, particularly selective laser melting (SLM)/laser powder bed fusion (LPBF) [21,47,48,49,50,51,52,53], which implies the layer-by-layer synthesis of powder material according to the computer-aid-designed (CAD) sliced model and provides the manufacturing of net-shape parts. As ceramic particle-reinforced composites are difficult to machine [5], applying near-net-shape production techniques are required, making SLM suitable and promising in relation to Al-based alloy composites with Al_2_O_3_ reinforcing.

SLM is characterized by the following set of main parameters:

*P*—laser power, W;
*V*—scanning speed, mm/s;
*t*—layer thickness, µm;
*h*—hatch spacing, µm.

Some more specific parameters, e.g., laser spot diameter and profile, substrate heating, and scanning strategy, result in the following sets of energy density (ED):

(1) $$\mathrm{LED}=\frac{P}{V}$$

where LED is the linear ED.

(2) $${\mathrm{ED}}_{a}=\frac{P}{Vh}$$

where ED_a_ is the aerial ED.

(3) $${\mathrm{ED}}_{V}=\frac{P}{Vht}$$

where ED_V_ is the volume ED.

ED is a simple way for the elementary comparison of different SLM regimes being useful and suitable, especially within working with specific SLM machines. A comparison of Eds used on different SLM machines should be made with the machines’ features considered.

In relation to MMC and taking into account those discussed above, the SLM approach has advantages due to its melting peculiarities. The small size of the molten pool, the extremely short lifetime of the liquid state, and, thus, the rapid heating and cooling rates are inherent in the SLM procedure. Therefore, superfine microstructures are usually obtained, which, in turn, leads to a higher strength and hardness according to a dispersion hardening mechanism and Hall–Petch theory [54,55,56,57,58,59]. Moreover, the finer reinforcement powder is used in the shorter distance between the reinforcing particles in the material, creating more barriers for the movement of dislocations [54]. Thus, the uniform reinforcing particle distribution is still needed to be achieved after the SLM process. The problem of ceramic particles segregation arises because of their rejection by the solid–liquid interface during solidification [5,60]. As the size of the molten pool is small and the cooling rates during SLM are extremely high, the particle segregation tendency would be not so significant. Moreover, this problem could almost be solved via SLM if laser irradiation produces an intensive melt flow [61], providing a uniform dispersion of ceramic particles subject to optimal SLM parameters. In addition, the direct impact of laser irradiation and the intensive heating of the small amount of powder material leads to enough heating of both aluminum and alumina particles, improving the wettability of the latter by the liquid Al matrix [62] and enhancing the bonds at the interface [29]. 

Generally, all the problems that also arise during the SLM process, namely, wettability, the heterogeneous distribution of particles, porosity, and the agglomeration particles, could be overcome by the thorough selection of SLM parameters and the initial mixing regime of powder materials. In summary, SLM combines the advantages of both PM and casting methods; thus, a thorough consideration of this method applied to Al-Al_2_O_3_ composites is of current scientific and technological interest. The current review aims to show the main achievements in this direction, to discuss the difficulties and possible ways to overcome them, and to compare SLM with conventional methods applied to AMCs with alumina. In particular, various approaches of initial powder preparation, SLM process regimes, material microstructure formation depending on the regimes, melting pool behavior, and mechanical properties of the composites are discussed. Scattered information is collected within the present review to help researchers in this field achieve better mechanical behavior results of Al-Al_2_O_3_ compounds fabricated via SLM.

## 2. Initial Powder Preparation

Mechanical alloying as a solid-state powder processing technique has attracted research interest [63] to prepare initial mixed Al-Al_2_O_3_ powder by means of ball-milling with a ball-to-powder weight ratio of 10:1 and the following regime: 16 h of milling with an interval of 10 min after each 30 min of milling. The ball-milling technique has some indisputable advantages applied to thorough mixing and milling, with repeated deformation, fracturing, and cold-welding providing the transfer of mass between components and a uniform distribution in the reinforcement case [64]. However, it should be noted that the application of intensive mechanical milling (ball milling) leads to numerous problems for subsequent selective laser melting. Namely, the milling of initial powder with a spherical morphology inevitably spoils the sphericity of the particles regardless of the ball-to-powder ratio, rotation speed, powder properties, particle size, etc. Moreover, the agglomeration and aggregation of particles with the expansion of particle size distribution during milling occurs especially in the case of nanosized particles [65]. Despite the suggestions in [63], all these could significantly decrease the powder flowability, which is of great importance in the formation of each layer during the SLM procedure, making the preparation of suitable composite powders challenging [66]. It is known [20,67] that a low flowability and sphericity, along with a wide particle size distribution, negatively affects selective laser melting, causing a decrease in powder bulk density, and resulting in a high porosity of the printed material. Another undesirable effect of ball milling that usually occurs is powder contamination (by iron, as a rule) of the material as a result of the abrasion and destruction of balls and camera walls [64]. Another peculiarity of the feedstock powder morphology in [63] is the ratio of the initial Al and Al_2_O_3_ particle size: an Al average particle size an order smaller than that of alumina, which is not typical for powders for the fabrication of metal matrix composites by means of any technique. The final particle size of Al_2_O_3_ particles after milling was significantly refined but remained more than twice as large. The features of this work [63] mentioned above could make a significant impact on the microstructure and mechanical properties, leaving room for further improvement in the mechanical behavior of Al-Al_2_O_3_ composites fabricated by SLM.

Despite that, the ball-milling method is widely used for SLM powder preparation. Han et al. [66,68] investigated ball-milled Al-Al_2_O_3_ nanocomposites for SLM applications, and they carried it out with 200 g of Al and 4 vol.% of Al_2_O_3_ powders with a 5:1 ball-to-powder weight ratio. Unlike Jue et al., the Al_2_O_3_ particle size in [66,68] was far less (<50 nm). Undesirable processes such as cold-welding or oxidation that are usually observed during ball-milling require special approaches. Han et al. offered the addition of stearic acid to prevent the cold-welding of the material and iron from the grinding bowl and balls, and the filling of the grinding bowls with argon gas to avoid oxidation. However, obtaining a high sphericity of the powder particles after ball-milling is still impossible, which, again, proved the powder images in the studies by Han et al. They also made a similar conclusion to Jue et al. about the combination of the milling for 10 min with pauses for 15 min in order to obtain a narrow particle distribution. It is well known [69,70,71,72,73,74,75] that achieving a proper mixing of nano-sized powders is challenging because of the tendency of the nanoparticles to agglomerate due to van der Waals forces. That is why the handling of nanopowders using simple mixing methods, for example, a tilting drum blender, is not applicable and needs more complex approaches, one of which is ball-milling. Du et al. [76] also reported the preparation of Al-based composites with 2 and 5 wt.% of nano-sized Al_2_O_3_ for SLM. Gas-atomized AlSi10Mg powder with normally distributed particles from 20 to 63 µm with a mean diameter of 42 µm was successfully mixed with the nano-sized nAl_2_O_3_ reinforcement powder (nominal diameter of 270 nm) by means of ball-milling for 5 h at 600 rpm and with a ball-to-powder ratio of 1:1. 

The advanced method of aluminum-alumina powder for SLM was proposed in previous studies [77,78,79]. The composite powder was obtained by the oxidation of aluminum in water, resulting in a core–shell aluminum-alumina morphology, wherein the sphericity and particle size distribution remained nearly unchanged, providing a high flowability and bulk density, which is of great importance for the further SLS/SLM process. The alumina content dependance on the temperature of the oxidation process was shown. 

**Figure 1:**
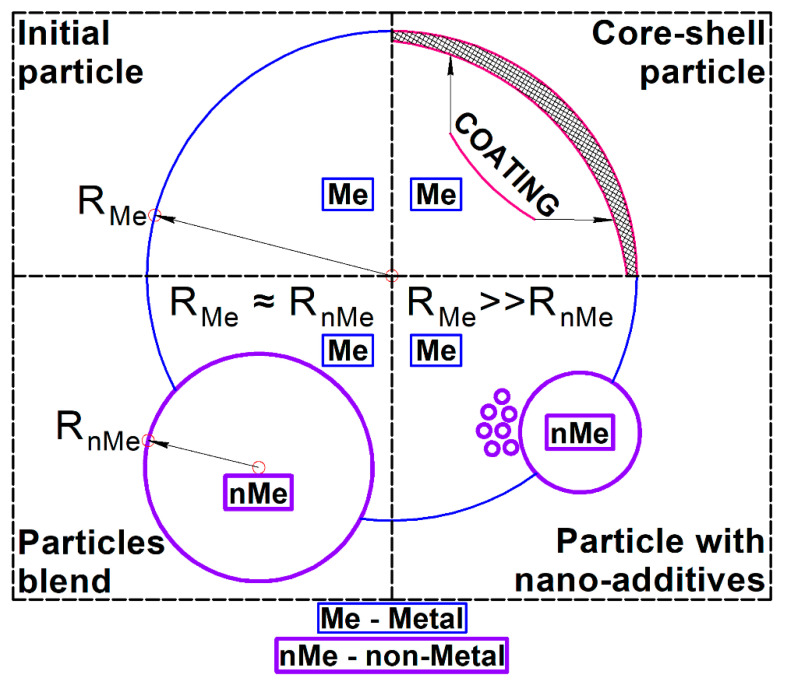
Types of initial powder morphology for SLM of metal-matrix composites with nonmetal additives.

Thus, the initial powder morphology could be divided into three main types: particle blends with comparable sizes of metal matrix particles and nonmetal additives; a mixture of nano-sized additives; and a powder of core-shell particles—see Figure 1.

The main features of the initial powder preparation, including the powder morphologies and mixing regimes used in the observed studies, are collected in Table 1.

## 3. SLM Regimes, Process Window, and Microstructure

The primary definition and optimization of the SLM parameters route for any new material is generally the same and represents a narrowing of the processing parameters window. The ED may change through variations in the laser power P or scanning speed v. Laser power has a directly proportional effect on the ED value, whereas the scanning speed has an inverse proportion, i.e., the higher the speed, the lower the energy supplied to the material per unit time. It is also worth noting that a lower temperature of the melting pool implies a poor viscosity of the melt and, hence, a poor wettability of the previous printed layer and Al_2_O_3_ particles suspended in the melt. Overall, too high a scanning speed leads to a discontinuous melting process, forming unmelted regions, pores, and cracks. Therefore, the densification rate of the material after such laser melting will not be high enough.

**Figure 2:**
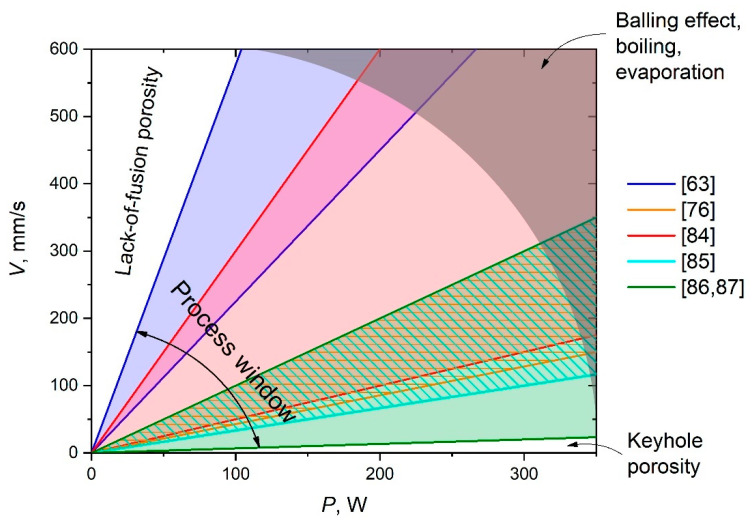
P–V diagrams representing process windows for Al-Al_2_O_3_ composites synthesis.

On the contrary, low scanning speeds increase the heated area and melting pool temperature, possibly leading to its boiling and, in turn, intensive evaporation and spattering. This not only results in pore formation but also causes undesirable chemical and phase transformations, the shrinkage of porosities, and the formation of thermal cracks [63]. In addition, this route of optimization of SLM parameters should be performed not only in new material development cases but also for each SLM machine, because of differences in their characteristics and features. Process windows for the synthesis of Al-Al_2_O_3_ composites used in various studies are depicted in Figure 2 as ranges of P–V ratios. Rays of the same P–V ratio indicate the same LED; regions between boundary rays of the same colors indicate the ranges of the used LED in the current research. The overall window includes the wide range of P–V ratios in which the tightly overlapped region is clearly seen. An insufficient laser power as a rule leads to the formation of unmolten defects (lack-of-fusion porosity region in Figure 2). Too slow a scanning speed, in turn, leads to keyhole porosity, while too high an ED initiates the balling effect [88,89], as well as boiling and evaporation of the melt (discussed in detail in Section 4). It does not mean that optimal SLM regimes must lie within the process window; on the contrary, an optimal P–V ratio could even go out of the frame of the overall window shown because of other factors such as powder morphology, layer thickness, hatch spacing, and scanning strategy. All boundaries of the mentioned regions are smooth. However, Figure 2 displays the most possible optimal window that one can focus on to start the optimization of the SLM parameters route. It should be mentioned that although such a direct comparison of the SLM regimes as in Figure 2 is not completely correct, because of other parameters and the difference in machine features, all reviewed research of Al-Al_2_O_3_ composites was performed using similar continuous-wave ytterbium fiber lasers of various powers with a TEM00 laser operation mode [90].

Such a route can be clearly seen in a previous study [63] applied to Al-Al_2_O_3_ composites. Densification levels from 80.6% up to 97.3% were obtained depending on the variation in scanning speed. The best result with a high relative density (RD) corresponded to a v of 550 mm/s (LED = 236 J/m), which is reasonable and optimal within the discovered processing window. Lower or higher scanning speeds decrease the densification level significantly. It should be recalled that a high volume of unmelted Al_2_O_3_ regions on the depicted microstructures and a reduced densification level could be partly explained by the feedstock powder morphology features mentioned above. It is worth noting that a simple linear scan pattern was employed in this study, whereas many recent studies [91,92,93,94] showed complex strategies (such as a 67° rotation between layers and chessboard) to be more effective at obtaining a high density and porous-free material. 

In a study by Han et al. [83], a Renishaw AM250 SLM machine was employed to print 8 × 8 × 8 mm^3^ cubic samples, and a striped fill-hatch-type scanning strategy was performed with the 67° rotation between each layer. A laser power of 200 W and a scanning speed of 300 mm/s (ED_V_ = 317.5 J/mm^3^) were found as optimum SLM parameters to synthesize the material with a high density of 99.49% [84]. Du et al. [76] reported the SLM of the AlSi_10_Mg and nAl_2_O_3_ composite on the SLM 250HL machine with an 80 µm laser spot size, a layer thickness of 0.05 mm, a laser power of 350 W, a scanning speed range of 100–1500 mm/s, and a 0.1–0.2 mm hatch spacing, providing an ED_V_ varying from 35 to 150 J/mm^3^. The chessboard island strategy with a 67° rotation was employed. The authors found that the use of a higher ED_V_ (109–175 J/mm^3^) is appropriate for dense samples of the AlSi_10_Mg-nAl_2_O_3_ composite, explaining it by the insulation of AlSi_10_Mg particles via the nAl_2_O_3_ layer. In addition, an increase in composite melting pool viscosity was observed, leading to porosity defects due to the weak inter-line bonding or discontinuous melting process. 

Balling phenomena [89], leading to poor interline bonding and gaps at low ED_V_, were observed. A higher ED_V_ induced the porosity formation that Du et al. [76] attributed to the increased recoil pressure. Moreover, it resulted in a higher unstable melt flow and melt splashing induced by both the Marangoni convection and the recoil pressure, along with the vapor intensification [95].

Besides this, Du et al. was faced with the dynamic keyhole process, leading to pore formation during rapid solidification, inherent in the aluminum alloy with elements of low boiling points such as Si, Mn, and Mg [96]. The decrease in RD was determined not only by porosity but also by crack formation. Du et al. observed thermal shrinkage and cracks in solidified alumina along with small cavities at the edge of the nAl_2_O_3_ after the low ED_V_ SLM process. According to [63,84], an increase in ED_V_ improves the particle–matrix interface.

The SLM of the Al-Al_2_O_3_ core–shell composite with 10 wt.% of alumina prepared by the special technique described in [77] was performed by authors of a previous study [78]. The ED_a_ of 7–8 J/mm^2^ was found to be the optimal range for the SLM of Al-10 wt.% Al_2_O_3_ core–shell powder composites, whereas the lower and higher energies caused an incomplete melting and aluminum boiling, respectively. Moreover, a lower ED insufficient for Al_2_O_3_ melting led to the agglomeration at molten pool boundaries and weak bonding between the matrix and reinforcement due to the γ-Al_2_O_3_ to α-Al_2_O_3_ phase transition. On the contrary, the ED_a_ increased beyond the 8 J/mm^2^ activated deoxidation and boiling of the molten pool processes. Both incomplete melting and boiling reduced the density. The employment of ED_a_ = 7.69 J/mm^2^ with *P* = 220 W, *h* = 0.13, and *V* = 220 mm/s led to the highest sample hardness (58.3 ± 0.9 HB) and RD (96.5%). Such a relatively low RD was explained by the authors by the γ- to α-Al_2_O_3_ phase transition and the particle aggregations. Further research into the double-layer exposure of the SLM mode is needed to overcome these problems. The already used and studied SLM regimes in detail are presented in Table 2.

**Figure 3:**
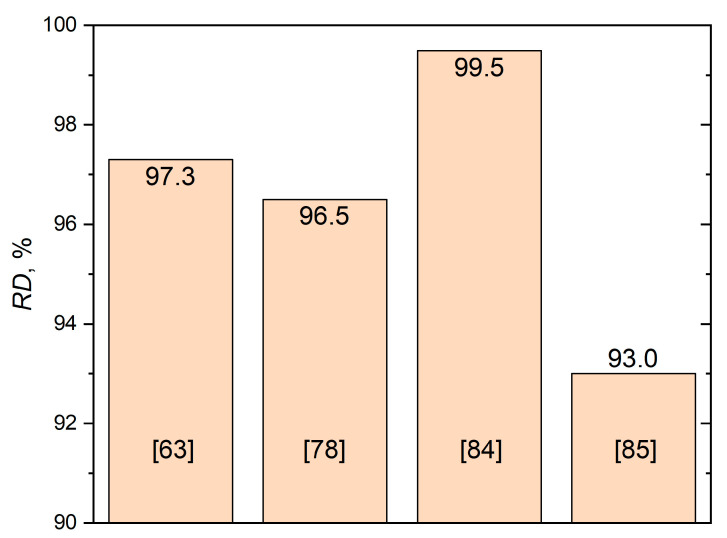
Maximum RD values of AMC + alumina materials achieved by means of the SLM technique.

The casting technology proposed in [33] allowed a porosity of 1.18% to be obtained for the composite with 1% of Al_2_O_3_. The porosity increased with Al_2_O_3_ content and reached more than 6% for a maximum alumina concentration of 10%. With such a complex route as stir casting with heat treatment, the addition of heat-treated particles to the melt by an inert argon gas flow could not provide a low porosity of at least less than 1%. In a previous study [41], relatively high values of RD (about 0.97) were reached only for 1 vol.% of nano-alumina after at least 150 min of sintering. The increase in both the volume fraction and particle size of alumina drastically decreased the RD. This showed the great complexity of melting and sintering approaches to produce fully dense Al-based matrix composites with Al_2_O_3_ reinforcement, wherein RD < 0.99 is usually considered unsatisfactory in the SLM case. The main results of the relative densities of SLMed AMCs with Al_2_O_3_ achieved in various studies are shown in Figure 3.

It should be mentioned that there is another indirect way to obtain the Al-Al_2_O_3_ composite described in [97,98]. The initial mixture of Al and 5 wt.% Fe_2_O_3_ was SLMed at a laser power of 40–90 W and a scanning speed of 70–140 mm/s. The heating of the material during the melting initiated the following reaction:

8Al + 3Fe_2_O_3_ → 2Fe_3_Al + 3Al_2_O_3_ + heat (4)

Eruptive sparks were observed during the process because of the extra heat that contributed to powder melting and molten pool extension. The fine particles with a uniform distribution in the Al matrix were defined as alumina and phases of Al, Fe, and O acting as reinforcements. 

Thus, it was shown that the SLM process allows the synthesis of the particles-reinforced AMC by in situ reaction. Unfortunately, there is no information about RD or any mechanical properties in that study, so it is impossible to compare the produced material with analogs.

## 4. Melting Pool Behavior

Processes within the melting pool during selective laser melting run extremely fast; hence, it is of great difficulty to study them. Some conclusions could be formulated based on the microstructural features after solidification. Jue et al. [63] showed the formation of agglomerated regions or a long-strip or ring morphology of Al_2_O_3_ particles because of their displacement to the boundaries of the melting pool. The exact character of such a structure depended on the energy density of the SLM, but generally, it showed a far-from-desirable microstructure with a nonuniform distribution of reinforcing particles in the composite, which prevented the achievement of high mechanical properties (discussed in the next section). Jue et al. [63] claimed that the complete melting of the initial powder occurs, including the Al_2_O_3_ phase and its formation during melting runs through the dissolution/precipitation mechanism. However, there are reasons [99,100] to believe that lower energy densities within the process window of Al-based materials via SLM do not correspond to temperatures above ~1600–1800 °C, which is lower than the alumina melting point (2044 °C); hence, the complete melting of the Al_2_O_3_ phase during SLM could be achieved only at elevated energy density levels. High EDs sufficient for the melting of Al_2_O_3_ could also be accompanied by undesirable processes of aluminum melt boiling (Al boiling temperature is 2519 °C), and its evaporation and emission. There are a series of studies about alumina loss during the Al-Al_2_O_3_ SLM process [86,87] that shows a temperature of the melting pool higher than the alumina melting point at any set of investigated SLM parameters. If the temperature of the molten pool is strongly influenced by the process parameters, the Al_2_O_3_ loss phenomenon may also be controlled by the SLM regime: the setting temperature of the molten pool above the Al_2_O_3_ melting point leads to alumina melt and resolidification; setting it above the boiling point (2977 °C) leads to alumina vaporization and loss [86]. The boiling point of Al is 2519 °C, so in the case of the temperature of the molten pool being higher than the alumina boiling point, Al will intensely burn. Thus, the presence or absence of Al loss during melting may be a marker of the molten pool temperature. In fact, no Al loss was usually observed, which means a temperature of the molten pool below 2500 °C, thus being far from the alumina boiling point. Liao et al. concluded that a notable Al_2_O_3_ loss could not be explained by vaporization. According to Laurent et al. [62], deoxidization by the dissociative vaporization of alumina becomes noticeable only at temperatures much higher than the melting point of aluminum. Some disruption of the alumina surface could be possible in the vicinity of the Al melting point; however, it only affects a film of a few monolayers in thickness. Besides the laser power and scanning speed, hatch spacing also affects the Al_2_O_3_ loss—the higher the hatch spacing, the lower the Al_2_O_3_ loss. However, this dependence was observed to be nonlinear [86]—the Al_2_O_3_ loss at a hatch spacing of 0.06 mm was smaller than that at a hatch spacing of 0.08 mm. It was explained by the difference in the laser absorptivity of the solid and powder [101], thus decreasing the temperature of the molten pool at higher overlap. According to [87], the main loss mechanism of Al_2_O_3_ is the reduction in the reaction of Al_2_O_3_ by aluminum. A critical temperature of 1520 °C was in the case of the Al_2_O_3_-AlSi_10_Mg composite, so it was lower than the usually observed molten pool temperature under laser irradiation. Thus, notable Al_2_O_3_ burning loss can be expected during the SLM and should be taken into account.

Returning to the temperature of the molten pool, there are some studies about the SLM/SLS of the Al_2_O_3_ ceramic [102,103] that showed the LED values normalized to the square of the laser spot to be of the same order as those for Al-based materials melting, making the consideration about the state of alumina particles within the melting pool highly controversial. At the same time, it should be noted that, first, any discussion about reinforcing the particle distribution within the melting pool involves the presence of those particles in the solid state and, secondly, extremely high heating and cooling rates with a short lifetime of the melting pool somewhat obstruct the complete melting of Al_2_O_3_. Therefore, it is possible to make an assumption that the behavior of Al_2_O_3_ particles in the melting pool may be considered as suspended solids within liquid. Based on this, the wettability of alumina by molten aluminum should be clarified. Laurent et al. [62] studied the problem and found that the contact angle θ smoothly decreased within the range from 933 (θ = 103 ± 6°) to 1273 K (θ = 86 ± 6°), which means that the higher the temperature of the melting pool, the better the wettability.

The segregation of Al_2_O_3_ particles during solidification is another phenomenon that should be discussed. This phenomenon arises because the Al_2_O_3_/solid-Al interfacial energy is higher than the Al_2_O_3_/liquid-Al energy [104,105], and the difference acts as a driving force, pushing alumina particles into the melt [106,107,108]. The so-called “particle pushing” results in the redistribution or segregation of the Al_2_O_3_ particles into regions that are last to solidify. 

**Figure 4:**
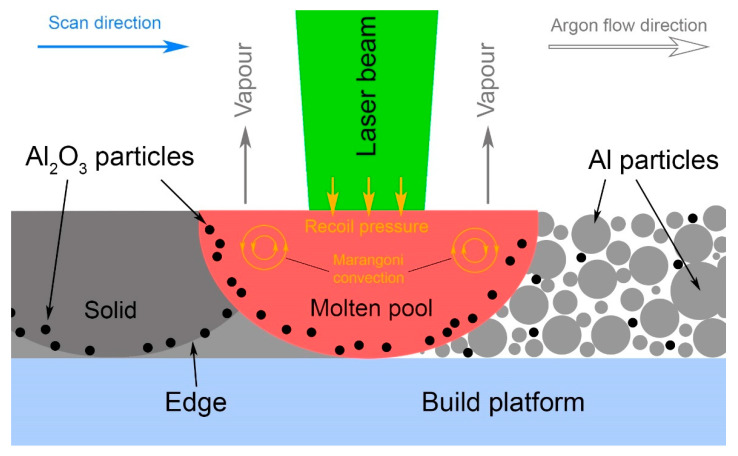
Schematic representation of reinforcing particles distribution and agglomeration along the melt track edges.

Extremely high cooling rates inherent in SLM allow this phenomenon to be successfully avoided and allow a uniform distribution of reinforced particles to be obtained, as it was accomplished on the powder mixing/milling stage. Jue et al. [63,80] also considered the distribution of alumina in the Al matrix specifically for SLM, taking into account the Marangoni convection and induced liquid capillary forces that accelerate the particle rearrangement, making it possible to obtain a homogeneous structure after SLM. Han et al. [84] studied the melting pool temperature during SLM with a 200 W laser power. It was observed that melting at a low scanning speed of 200 mm/s could provide the heating of the melt above the alumina melting point (2040 °C). Du et al. [76] claimed that even a higher energy input and the melting of at least some part of the nAl_2_O_3_ could result in the increase in the nAl_2_O_3_ particle size. In the studies of both Du et al. and Han et al., Al_2_O_3_ particle agglomerations were observed along the melt track edges (see Figure 4), which was explained by reinforcing particles pushing outward due to Marangoni convection and the recoil pressure.

## 5. Mechanical Properties

Besides the variations in feedstock powder and SLM parameters, every SLM machine has unique features such as laser spot diameter and configuration (profile). That is why the direct comparison of SLM regimes of even the same material fabrication by means of different machines is unreasonable, wherein the mechanical properties of printed samples are comparable and reflect whether or not the optimal regime has been selected. However, the diversities of the investigated mechanical characteristics and measurement methods make comparison difficult, especially when dealing with SLMed materials. There is a lack of data on mechanical properties in the literature on the topic of SLM of Al-Al_2_O_3_ composites. Despite this, some information and discussion are offered below.

The main goal of aluminum or its reinforcing alloys is enhancing their mechanical properties, especially hardness, strength, and wear resistance. The synthesis of Al-Al_2_O_3_ composites by SLM also pursues this goal, and remarkable results have been achieved. As far as Al_2_O_3_ being a ceramic with high hardness, tribological properties, and good performance at elevated temperatures [109], it was successfully employed as a reinforcing phase in Al alloys, improving their mechanical behavior due to being an obstacle for the movement of dislocations [54]. SLM is one of the advanced and promising methods of AMCs production due to, first, the freedom in the part design and the absence of the necessity of machining, and, secondly, a specific ultrafine microstructure after SLM [55,56,57,58,59]. The fine microstructure provides grain-boundary strengthening (or Hall–Petch strengthening), leading to an enhanced strength of the SLMed material. Thus, it is not surprising that the fabrication of AMCs by means of the SLM is of great scientific interest. The mechanical properties of the SLMed composite materials are presented in Table 3, and they were compared with analogs fabricated via the conventional method such as casting and sintering. Al_2_O_3_ reinforcement significantly increases the hardness of Al. Jue et al. [63] obtained high microhardness values of 148–175 HV0.1 depending on the SLM parameters, along with superior wear performance, namely, a coefficient of friction COF of 0.11 and a wear rate of 4.75 × 10^−5^ mm^3^ N^−1^ m^−1^. Detailed investigations of the surface roughness, COF, and wear rate of Al-Al_2_O_3_ SLMed composites were carried out by Jue et al. in [81,82]. A series of studies by Han et al. [83,84] on the fabrication of Al-Al_2_O_3_ composites via SLM also concerned the microhardness, wear behavior, and COF. Enhancements of the macroscale wear behavior and microhardness, along with the increase in frictional coefficient, were discovered. Alumina addition led to a 17.5% increase in microhardness. Moreover, essential information about the tensile behavior was presented in [84]. A significant improvement of 36.3% (from 80 to 109 MPa) in yield strength compared with pure aluminum produced through the same regime was proposed. Ultimate tensile strengths (UTSs) of the composite material and pure Al of 160 and 110 MPa, respectively (45.5% improvement), were found. The UTS value of 160 MPa is still relatively low compared with AMCs fabricated by some conventional technologies [32,110], though it exceeds some other results obtained on Al-Al_2_O_3_ composites [5,111]. This implies that the further mechanical properties of the improvement of Al-Al_2_O_3_ MMC should be soon expected by more precise SLM process optimizations.

## 6. Conclusions

Al-Al_2_O_3_ composites are still of great importance as high-performance materials for various industries due to the combination of excellent mechanical and wear properties with low weight. As far as the machining of ceramic reinforced composites associated with significant difficulty, near-net-shape fabrication techniques such as SLM are attracting increased attention. Both SLM and conventional techniques (casting, sintering, etc.) are faced with similar issues: increasing the density of the material (avoiding pores, cracks, etc.) and obtaining a homogeneous distribution of reinforcing particles within the matrix. There is significant progress in the production of Al-Al_2_O_3_ composites by means of SLM. Nearly fully dense composite materials (RD > 99%) are relatively easy to obtain via SLM using optimal processing parameters, avoiding the formation of defects. Initial powder preparation plays a crucial role in determining the density and microstructure; the ball milling method was shown as the most verified and commonly used for now, but with drawbacks such as the deterioration of particle sphericity and expansion of the particle size distribution. Alternative approaches such as the oxidation of aluminum in water resulting in a core–shell morphology or the formation of in-situ Al–(Al_2_O_3_ + Fe_3_Al) composites during melting of the Al and 5 wt.% Fe_2_O_3_ powder are devoid of ball milling shortcomings but require more experiments for verification. The Al and 4 vol.% of the Al_2_O_3_ sample with the highest density among all reviewed data were obtained using *P* = 200 W, *V* = 300 mm/s, and a layer thickness of 30 µm on the Renishaw AM250 machine. The distribution of reinforcing particles within the molten pool and the final structure is still challenging. Particulates tend to consolidate near molten pool/grain boundaries being pushed by the liquid–solid interface during the solidification process, not allowing higher mechanical properties to be achieved. Increasing the ED enough for Al_2_O_3_ melting with its subsequent precipitation seems to be a way to synthesize the Al-base matrix composite with uniform alumina distribution, wherein the excessive increase in ED and melting pool temperature leads to Al boiling and, therefore, to the formation of extra defects in the structure. Thus, the precise optimization of SLM parameters has already allowed a uniform distribution to be obtained at least along the boundaries without huge agglomerations, and it should allow further improvements in the mechanical properties. There is still a lack of experimental data about the tensile behavior of these materials after SLM. Therefore, based on the reviewed research and data, there are great prospects for the further research of Al-Al_2_O_3_ composites fabricated via SLM.

## Figures and Tables

**Table 1:** Feedstock powder characterization.

Material	Powder Morphology	Mixing Regime	Reference
Al-Al_2_O_3_ 80:20 weight ratio	Al—1.02 µm average particle size; Al_2_O_3_—9.04 µm.	High-energy planetary mono-mill. 16 h with interval of 10 min after each 30 min of milling. 200 rpm.	[63]
AlSi_10_Mg-Al_2_O_3_ 80:20 weight ratio	AlSi_10_Mg—gas atomized, 30 µm average particle size; Al_2_O_3_—9.04 µm.	Ball-to-powder weight ratio of 10:1, a rotation speed of 200 rpm, and a milling time of 8 h.	[80,81,82]
Al and 4 vol.% of Al_2_O_3_	Al—17.1 µm average particle size; Al_2_O_3_—10.37 µm.	Ball-to-powder weight ratio of 5:1, 350 rpm, 20 h. Milling of 15 min and pause of 5 min (method 1), milling of 10 min and pause of 15 min combination (method 2)	[66,83,84]
AlSi_10_Mg + 2–5wt.% nanoAl_2_O_3_	AlSi_10_Mg—atomized, 42 µm average particle size; nAl_2_O_3_—270 nm	Single-axis ball milling at 600 rpm, 5 h, ball-to-powder ratio of 1:1 in a volatile solvent	[76]
Al_2_O_3_-AlSi_10_Mg 1:1 weight ratio	AlSi_10_Mg—33.1 µm average particle size; Al_2_O_3_—26.6 µm	Tumbling ball mill, weight ratio of 1:1	[85]
Al_2_O_3_- AlSi_10_Mg 15:85 weight ratio	AlSi_10_Mg—33.1 µm average particle size; Al_2_O_3_—26.6 µm	10:1 ball-to-powder weight ratio. 4 h, 70 rpm.	[86,87]
Al + 10 wt.% Al_2_O_3_ core–shell	D_50_ = 42 μm with narrow SPAN (D_90_ − D_10_)/D_50_ = 1.1)	Core–shell Al-Al_2_O_3_ powder obtained by hydrothermal oxidation	[78]

**Table 2:** SLM parameters and RD.

Material	SLM Parameters	Maximum RD, %	Reference
Al-Al_2_O_3_ 80:20 weight ratio	Spot size = 70 µm, linear raster scan pattern, *P* = 130 W, *V* = 450, 550, 650, 750 mm/s, LED = 173, 200, 236, 289 J/m	97.3	[63]
Al and 4 vol.% of Al_2_O_3_	Renishaw AM250, preheated T = 170 °C, *P* = 200 W, *V* = 100–600 mm/s (300 is optimal), *t* = 30 µm *h* = 70, 100 μm.	99.49	[84]
AlSi_10_Mg + 2–5wt.% nano-Al_2_O_3_	SLM 250, chessboard island strategy with a rotation of 67° at every layer. *P* = 350 W, *V* = 100–1500 mm/s, *h* = 0.1–0.2 mm. ED_V_ = 35–150 J/mm^3^ (109 is optimal)	–	[76]
Al_2_O_3_-AlSi_10_Mg 1:1 weight ratio	SLM-100, *P* = 200 W, *V* = 150, 200, 250, 300, and 350 mm/s(250–350 are optimal), *t* = 20 µm, *h* = 0.05, 0.1 µm (0.1 is optimal)	93	[85]
Al_2_O_3_-AlSi_10_Mg 15:85 weight ratio	*P* = 200 W, *V* = 100–300 mm/s, *t* = 20 μm, *h* = 60–160 μm	–	[86,87]
Al + 10 wt.% Al_2_O_3_ core–shell	SLM 280 HL, *P* = 220 W, *V* = 220 mm/s, EDa = 7–8 J/mm^2^, *h* = 0.13	96.5	[78]

**Table 3:** Mechanical properties of Al-Al_2_O_3_ composites fabricated by various methods.

Material	Method	Hardness	Mechanical Properties	Reference
Al-Al_2_O_3_ 80:20 weight ratio	SLM	175 HV0.1	COF = 0.11, wear rate = 4.75 × 10^−5^ mm^3^ N^−1^ m^−1^	[63]
AlSi_10_Mg-Al_2_O_3_ 80:20 weight ratio	SLM	-	COF = 0.3, wear rate = 2.94 × 10^−5^ mm^3^ N^−1^ m^−1^	[81,82]
Al and 4 vol.% of Al_2_O_3_	SLM	48.5 HV/0.1	Yield strength σ_0.2_ = 109 MPa, UTS = 160 MPa	[83,84]
Al + 10 wt.% Al_2_O_3_ core–shell	SLM	58.3 ± 0.9 HB	-	[78]
Al + 10wt.% Al_2_O_3_	Sintering	76 HB	Compressive strength = 318 MPa, maximum elongation = 61.8%	[4]
A356 alloy + 1–10 wt.% Al_2_O_3_	Stir casting	≤76.3 HB	Compressive strength ≤ 610 MPa	[33]
2024 alloy + 10–30 wt.% Al_2_O_3_	Stir casting	≤135 HB	Tensile strength up to 112 MPa, elongation below 3%	[5]
Al6061 + 0.5, 1, 1.5 wt.% nano-Al_2_O_3_	Stir casting	≤79 HB	UTS > 250 MPa	[110]
Al/Al_2_O_3_	Die casting, hot extrusion, and T-651 heat treatment	-	UTS = 372 MPa, YS = 352 MPa, elongation = 3–4%	[32]
Al + 5, 10, 15 vol.% Al_2_O_3_	Microwave sintering	≤92.65 HV	UTS ≤ 154 ± 6 MPa, YS ≤ 139 ± 8 MPa, elongation = 7.2%	[111]

## Data Availability

Data sharing is not applicable to this article.
